# Lightweight and efficient skeleton-based sports activity recognition with ASTM-Net

**DOI:** 10.1371/journal.pone.0324605

**Published:** 2025-07-08

**Authors:** Bin Wu, Mei Xue, Ying Jia, Ning Zhang, GuoJin Zhao, XiuPing Wang, Chunlei Zhang

**Affiliations:** 1 College of Light Textile and Chemical Engineering, Binzhou Polytechnic, Shandong, China; 2 School of Information Engineering, Binzhou Polytechnic, Shandong, China; 3 Youth League committee, Binzhou Polytechnic, Shandong, China; 4 Shandong Binzhou Vocational College, Shandong, China; National University of Defense Technology, CHINA

## Abstract

Human Activity Recognition (HAR) plays a pivotal role in video understanding, with applications ranging from surveillance to virtual reality. Skeletal data has emerged as a robust modality for HAR, overcoming challenges such as noisy backgrounds and lighting variations. However, current Graph Convolutional Network (GCNN)–based methods for skeletal activity recognition face two key limitations: (1) they fail to capture dynamic changes in node affinities induced by movements, and (2) they overlook the interplay between spatial and temporal information critical for recognizing complex actions. To address these challenges, we propose ASTM‑Net, an Activity‑aware SpatioTemporal Multi‑branch graph convolutional network comprising two novel modules. First, the Activity‑aware Spatial Graph convolution Module (ASGM) dynamically models Activity‑Aware Adjacency Graphs (3A‑Graphs) by fusing a manually initialized physical graph, a learnable graph optimized end‑to‑end, and a dynamically inferred, activity‑related graph—thereby capturing evolving spatial affinities. Second, we introduce the Temporal Multi‑branch Graph convolution Module (TMGM), which employs parallel branches of channel‑reduction, dilated temporal convolutions with varied dilation rates, pooling, and pointwise convolutions to effectively model both fine‑grained and long‑range temporal dependencies. This multi‑branch design not only addresses diverse action speeds and durations but also maintains parameter efficiency. By integrating ASGM and TMGM, ASTM‑Net jointly captures spatial–temporal mutualities with significantly reduced computational cost. Extensive experiments on NTU‑RGB + D, NTU‑RGB + D 120, and Toyota Smarthome demonstrate ASTM‑Net’s superiority: it outperforms DualHead‑Net‑ALLs by 0.31% on NTU‑RGB + D X‑Sub and surpasses SkateFormer by 2.22% on Toyota Smarthome Cross‑Subject; it reduces parameters by 51.9% and FLOPs by 49.7% compared to MST‑GCNN‑ALLs while improving accuracy by 0.82%; and under 30% random node occlusion, it achieves 86.94% accuracy—3.49% higher than CBAM‑STGCN.

## I. Introduction

Human Activity Recognition (HAR) aims to identify human activities based on the full execution of actions captured in videos, serving as a core task in video understanding [1]. It has already found widespread applications in daily life. For instance, activity recognition algorithms power visual surveillance systems that assist in capturing criminals through video analysis, reducing the risks of criminal activities [2]. Video retrieval enables users to search for videos matching textual descriptions (e.g., titles or keywords) on the internet [3]. Additionally, virtual reality (VR) technologies based on depth sensor data have attracted a large audience across all age groups in the gaming industry [4]. Existing research has explored various modalities for video feature representation, such as RGB frames, optical flow, and skeletal data. Among these, skelecton-based activity recognition has gained increasing attention in recent years due to advances in human pose estimation algorithms [5,6] and the robustness of skelecton data, which captures human activity information without being influenced by noisy backgrounds or variations in lighting [7].

With the development of deep learning, significant progress has been achieved in skelecton-based activity recognition using deep learning methods. Early approaches treated skeletal trajectories as either coordinate vector sequences or grid-like structures, inputting them into Recurrent Neural Networks (RNNs) [8] or Convolutional Neural Networks (ConvNets) [9]. However, RNNs and ConvNets are inherently designed for regular Euclidean data, and due to the irregular nature of skelecton data, these methods fail to fully exploit the affinities among nodes [8]. Graph Convolutional Neural Networks (GCNNs) [10], which excel in handling irregular data, have thus been introduced for modeling skelecton data.

For skelecton-based activity recognition, we transform skeletal data into a graph representation based on the physical connections between human nodes and encode the affinities among nodes using an adjacency matrix. On this basis, we define the implementation process of spatiotemporal graph convolution over the skelecton graph and skelecton trajectories. Finally, the spatiotemporal graph convolution is employed to extract skeletal features for human activity classification.

Although existing GCNN-based methods have demonstrated notable performance, most approaches alternate spatial graph convolutions and temporal graph convolutions independently [11–14]. This separation introduces two key limitations:

1) These methods neglect the dynamic changes in adjacency graph induced by node movements, failing to fully exploit activity information;
2) They overlook the mutualities between spatial and temporal information, which are critical for accurately recognizing skeletal activities.

Specifically, the impact of activity on node affinities can be summarized in two aspects:

1) Linkages between moving nodes: Nodes from the same body part exhibit synchronized movements due to inherent physical connections (e.g., nodes in the right upper limb during a “wave” activity). Similarly, mutualities between nodes from different body parts (e.g., both hands during a “clap” activity) indicate affinities caused by coordinated movements.
2) Variation in activity patterns across trajectories: Different skelecton trajectories demonstrate diverse activity patterns (e.g., speed or acceleration). Even when performing the same activity, individuals may exhibit unique activity patterns. Leveraging these rich activity patterns is essential for building more robust models.

Additionally, existing GCNN-based methods suffer from critical limitations in robustness and efficiency. These limitations stem from a fundamental disconnect between spatial graph learning and temporal dynamics, hindering both robustness to real-world variations and computational efficiency (These will be analyzed in detail in the later experimental sections).

To address these limitations, we propose an Activity Aware Spatial Graph Convolution Module (ASGM) to dynamically and efficiently model Activity Aware Adjacency Graph (3A-Graph). Specifically, the 3A-Graph, i.e., *G*, comprises three components:

1) A manually initialized graph *A* based on physical node connections.
2) A trainable graph *B*, optimized along with the network.
3) A dynamically inferred activity-related graph *C*, capturing the temporal affinity variations among nodes.

To learn *G*, we first employ an information extraction module to obtain semantic activity features. We then design a graph optimization module to infer *C*, which is used to refine *A* and *B*, ultimately generating *B*. Notably, ASGM learns the activity-related graphs across different time periods in a parameter-sharing manner, avoiding redundant parameters and improving modeling efficiency. By integrating activity information into spatial graph convolutions, ASGM achieves robustness against varying activity patterns and enables mutualities between spatial and temporal information. Additionally, by combining ASGM with Temporal Multi-branch Graph Convolution Module (TMGM), we construct an Activity Aware SpatioTemporal Multi-branch Graph Convolutional Neural Network (ASTM-Net) for skelecton-based activity recognition. Experimental results on the different datasets demonstrate that the proposed ASTM-Net outperforms State-Of-The-Art (SOTA) methods, validating its effectiveness.

The primary contributions of this paper are summarized as follows:

1) We propose a novel ASGM that dynamically models 3A-Graphs, capturing temporal node affinities and integrating them into spatial graph convolutions to enhance robustness against varying activity patterns.
2) We design a 3A-Graph framework comprising three components: an initialized graph based on physical connections, a trainable graph optimized during training, and a dynamically inferred activity-related graph. This framework enables the efficient representation of dynamic node affinities over time.
3) Building upon ASGM, we develop ASTM-Net, which integrates ASGM with the TMGM to model multi-scale temporal features effectively.
4) The proposed ASTM-Net achieves state-of-the-art performance with significantly fewer parameters and lower computational costs compared to existing methods, making it a lightweight and efficient solution for skelecton-based activity recognition.

## II. Related works

In the field of HAR, researchers have explored various modalities such as RGB images, optical flow, and skeletal data. Among these, the skeletal modality, which represents the human body topology with nodes and bones, demonstrates greater stability when dealing with complex environments, including variations in lighting, viewpoint, and speed. As a result, skelecton-based activity recognition has become a key area of focus in recent studies. With the rapid advancements in deep learning, methods leveraging ConvNets, RNNs, and GCNNsfor skelecton-based activity recognition have emerged. Table 1 summarizes the advantages and disadvantages of some representative approaches.

**Table 1:** Summary of the advantages and disadvantages of representative methods.

Methods	Representative Works	Advantages	Disadvantages
ConvNet-based	Li et al. [15], Xie et al. [16]	Efficient modeling of local node relationships using established convolutional structures.	Forcefully mapping non-Euclidean skeleton data into images loses global node relations; limited receptive field.
RNN-based	Kaseris et al. [17], Feng et al. [18]	Good at capturing long-term dependencies, suitable for continuous action modeling.	Weak spatial modeling capability, unable to effectively express complex topological relationships between nodes.
Spectral GCNNs	ST-GCNN [19], ChebyNet [20]	Theoretically rigorous, global feature aggregation through graph Fourier transform.	Relies on fixed graph Laplacian matrix, unable to model dynamically; high computational complexity.
Spatial GCNNs	DCNN [21], GAT [22]	Direct aggregation of spatial neighbor features, supports dynamic graph structures, computationally efficient.	Manually defined neighbor rules may ignore long-distance node dependencies (e.g., hand collaboration actions).
Dynamic Graph	DGCN-MPA [23], HAM-HGNet [24]	Dynamically adjusts node relationships, adapts to changes in actions (e.g., differences in movement speed).	Dynamic graph generation module increases the number of parameters, prone to overfitting small datasets.
Attention-enhanced	CBAM-STGCN [25], KDS-GCN [26]	Enhances the representation of key nodes/channels through attention mechanisms (e.g., importance of hand nodes in ‘throwing’).	Attention computation introduces additional overhead.
Hybrid Architectures	Wu et al. [27], Mansouri et al. [28]	Combines CNN (temporal) and GCN (spatial) to improve multimodal feature representation.	Dual-branch design leads to parameter redundancy, difficult to deploy on edge devices.
Transformer-based	SkateFormer [29], HCTransformer [30]	Self-attention mechanism captures global spatiotemporal dependencies, overcoming the locality limitation of graph convolution.	High computational complexity, large memory consumption for long sequences.

### A. Skelecton-based activity recognition with convnets

ConvNets have been applied to skelecton-based activity recognition using two main approaches. The first approach treats the raw keypoint coordinate data (i.e., *x*, *y*, *z*) as RGB channels of an image, mapping the skelecton data into a three-channel format and using ConvNet models for classification. Li et al. [15] proposed a dataset-independent and translation-scale-invariant mapping method, converting 3D skelecton data into color images with guaranteed invariance in scale. To fully extract skelecton features, Xie et al. [16] introduced a dual-stream ConvNet model that considers both node and bone features, employing asymmetric convolution blocks to mitigate deformation effects in skelecton trajectories. The second approach involves custom-mapping activity and temporal information from skelecton data into color images and classifying these withConvNets. Hożyń et al. [31] defined custom distance features based on skelecton data, mapping them into textured color images and training the model with AlexNet. To better incorporate temporal information, Younsi et al. [32] proposed Joint Trajectory Maps (JTMs), which encode spatiotemporal node trajectory information as three texture images from front, side, and top views for activity recognition.

However, ConvNet-based methods in skelecton activity recognition often simplify skelecton information into image representations, limiting feature learning to adjacent nodes within a convolution kernel [33]. This approach neglects latent linkages among all nodes.

### B. Skelecton-based activity recognition with RNNS

RNNs are adept at extracting long-term sequential information, making them suitable for processing skeletal activity data as temporal trajectories where node coordinates change over time. Kaseris et al. [17] partitioned the human skelecton into five parts and arranged the node coordinates into long vectors for input into Long Short-Term Memory (LSTM) networks for activity recognition. Recognizing that some activities only involve a few nodes, Feng et al. [18] proposed an end-to-end spatiotemporal attention network using RNNs, focusing on critical frames and key nodes. However, the spatial modeling limitations of RNNs generally prevent these methods from achieving competitive results [34].

### C. Skelecton-based activity recognition with GCNNS

In recent years, graph convolution techniques have advanced significantly, and skeletal data, inherently non-Euclidean, naturally aligns with graph structures where nodes represent nodes. Compared to ConvNets and RNNs, GCNNs can simultaneously model spatial and temporal affinities, drawing increasing attention from researchers. The core idea is to aggregate edge information to update node representations. GCNNs excel in learning from data with graph topologies and are well-suited for modeling non-Euclidean spatial data. GCNN methods are broadly classified into spectral-based and spatial-based approaches.

#### a. Spectral-based GCNNs.

**Fig 1:**
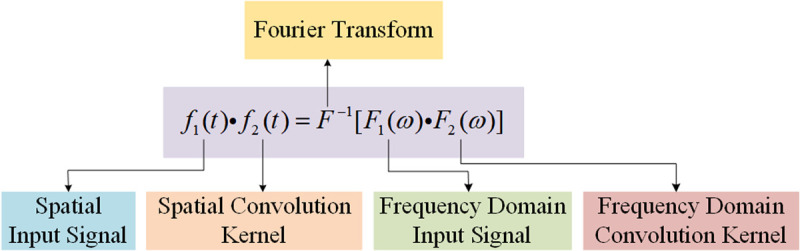
The system of spectral-based GCNNs.

Classical convolution kernels require fixed neighborhood sizes around the central node, which is challenging for graph-structured data due to variable and unordered node neighborhoods. Spectral-based GCNNs leverage graph signal processing, using Fourier transforms to convert graph signals into the frequency domain for convolution operations and then back to the spatial domain via inverse Fourier transforms [35], as shown in Fig 1.

Examples include the first-generation GCNN [10], Chebyshev Networks (ChebyNet) [20], and first-order approximations of ChebyNet [21]. The first-generation GCNN’s reliance on Laplacian matrix eigendecomposition leads to high computational costs, limiting practical use. To address this, ChebyNet approximates convolution kernels with Chebyshev polynomials, greatly improving efficiency. Xu et al. [36] further simplified ChebyNet with a first-order approximation, represented as:


$$H^{\left(l+1\right)}=\sigma ({\tilde{D}}^{-1/2}\tilde{A}{\tilde{D}}^{-1/2}H^{\left(l\right)}W^{\left(l\right)})$$ (1)

where $\tilde{A}=A+I_{N}$ is the adjacency matrix with self-loops, I_N_ is the identity matrix, $\tilde{D}$ is the degree matrix, W^(l)^ denotes the trainable weight matrix for layer l, σ(.) is the nonlinear activation function, and $H^{\left(l\right)}$ represents features at layer l with input H = X.

#### b. Spatial-based GCNNs.

Spatial-based GCNNs eliminate affinity on graph spectral theory, instead aggregating information from neighboring nodes to update central nodes from a spatial perspective [22,37]. These methods avoid Laplacian matrix constraints, enabling applications to directed graphs. Spatial-based GCNNs, akin to extended ConvNets, perform weighted summation of node information to achieve convolution [38]. Steps include propagating node features to neighbors, aggregating neighborhood features, and applying linear and nonlinear transformations to enhance model expressiveness. Examples include Diffusion Convolutional Neural Networks (DCNN) [26], Graph Sample and Aggregate (GraphSAGE) [39], and Graph Attention Networks (GAT) [40]. DCNN controls information flow via transition probabilities, GraphSAGE uses random sampling to aggregate features, and GAT employs attention coefficients to update node features, offering diverse methods for graph topology convolution. Recent advancements focus on enhancing spatial modeling through dynamic graph topologies and attention mechanisms, as follows:

1) Dynamic Graph Construction: Huang et al. [23] proposed dynamic graph convolutional networks with multi-scale position attention (DGCN-MPA), adaptively fusing hierarchical attention to adjust node relationships across scales. Yang et al. [24] designed a hierarchical adaptive multi-scale hypergraph (HAM-HGNet) that clusters joints into parts based on movement patterns, relaxing fixed topology constraints.
2) Knowledge & Attention Integration: Roy et al. [41] introduced knowledge-driven shift-GCN (KDS-GCN), leveraging graph connectivity priors and coordinate features to enrich representations. Qin et al. [25] enhanced ST-GCN with convolutional block attention modules (CBAM-STGCN), refining spatial-channel features through adaptive weighting.
3) Hybrid Architectures: To bridge Euclidean and non-Euclidean modeling, Wu et al. [27] combined multi-channel CNNs with GCNs, where CNNs capture local temporal patterns and GCNs extract spatial dependencies. Mansouri et al. [28] similarly fused CNNs and GCNs to jointly learn spatiotemporal dynamics.
4) Transformer-Based Extensions: Addressing GCNs’ limited receptive fields, Do et al. [29] proposed SkateFormer, partitioning skeletal-temporal relations for memory-efficient self-attention. Lin et al. [30] developed HCTransformer, decoupling human-centric and context features through domain-invariant interaction learning.

Furthermore, recent advances in graph neural networks across diverse domains have inspired novel skeleton-based HAR designs. For example, Su et al. [42] introduced MFEn-GFDn, a Multi-Feature Extraction Network and Graph Fusion Detection Network, to enhance radar target detection in complex sea clutter by constructing multi-feature graph data and applying graph fusion strategies. Zhang et al. [43] demonstrated the integration of GNNs with brain functional network analysis, highlighting the potential of interpretable GNN architectures for large-scale, heterogeneous graphs in clinical neuroscience applications. In the realm of multimodal video analysis, Wang et al. [44] proposed a Geo-based two-stream framework that builds facial graphs and fuses GCN and CNN features alongside temporal attention, achieving robust personality trait prediction in short video clips. These works underscore the versatility of GNNs in capturing complex relational patterns, motivating their application to dynamic skeleton activity graphs in HAR.

## III. Methodology

### A. Definitions

#### a. Construction of the skelecton graph.

Given a human skelecton, let *N* represent the number of nodes. The skelecton is naturally abstracted as a graph, where a skeleton with *N* nodes is represented as an undirected graph *G*=(*V*,*E*). Here, *V*={*v*_*i*_|*i* = 1,2,…,*N*} represents all vertices of the skelecton graph, corresponding to the nodes of the skelecton, and *E* represents the edges of the graph, corresponding to the physical connections between nodes. The affinity relationships between nodes are encoded into an adjacency matrix $\tilde{A}$ of size N*N, with elements defined as:


$${\tilde{A}}^{ij}=\left\{ \begin{array}{c} 1,(i=j)\;or\;(v_{i}\&v_{j}are\;connected)\\ 0,otherwise \end{array}\right.$$ (2)

#### b. Implementation of traditional graph convolution.

Based on the constructed skelecton graph, ST-GCNN (Spatiotemporal graph convolutional neural network) [19] defines the implementation of graph convolution on skeletal data. Given an input feature map $f_{in}\in R^{C_{in}*T*N}$, where *C*_*in*_ represents the number of input channels and *T* represents the number of frames, the process of graph convolution on skelecton data is expressed as:


$$f_{out}=\sum_{k=1}^{K}W_{k}f_{in}(A_{k}⊙L)$$ (3)

where *K* is the number of spatial subsets, set to 3 in ST-GCNN. $A_{k}=\Lambda_{k}^{-1/2}{\tilde{A}}_{k}\Lambda_{k}^{-1/2}$ represents the normalized adjacency matrix, and $\Lambda_{k}=\sum_{j=1}^{N}{\tilde{A}}_{k}^{ij}+\lambda$ is the normalized diagonal matrix, with *λ* = 0.001 to prevent empty rows in ${\tilde{A}}_{k}$. Note that $\sum_{j=1}^{N}{\tilde{A}}_{k}=\tilde{A}$. $W_{k}\in R^{C_{out}*C_{in}}$ is the trainable weight matrix, where *C*_*out*_ represents the number of output channels. $L\in R^{N*N}$ is a trainable matrix initialized with all elements set to 1. The operator ⊙ denotes the Hadamard product (element-wise multiplication). Using formula (3), the spatial graph convolution output $f_{out}\in R^{C_{out}*T*N}$ can be obtained.

### B. ASGM

**Fig 2:**
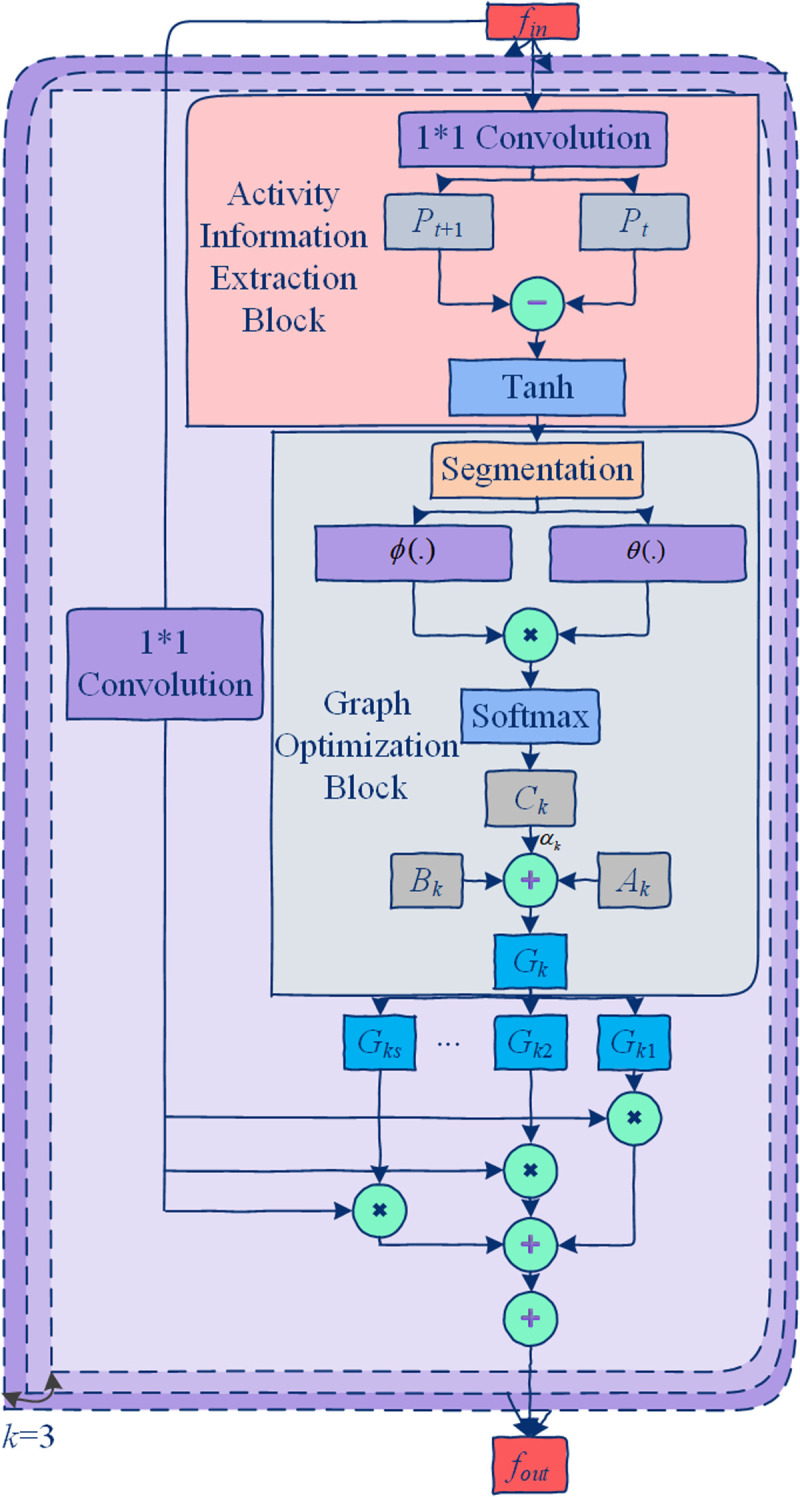
Overall framework of the proposed ASGM. The module extracts semantic features of activity information, dynamically infers an activity-related adjacency graph (3A-Graph), and then applies spatial graph convolutions using the optimized adjacency graph to model activity-specific spatial relationships.

The framework of the proposed ASGM is illustrated in Fig 2.

The process consists of three main stages:

1) Extract semantic features of activity information.
2) Dynamically infer a activity-related graph to capture the pairwise affinities among nodes across different time periods and optimize the skelecton graph.
3) Perform graph convolution using the optimized adjacency graph to obtain the final ASGM output.

**Fig 3:**
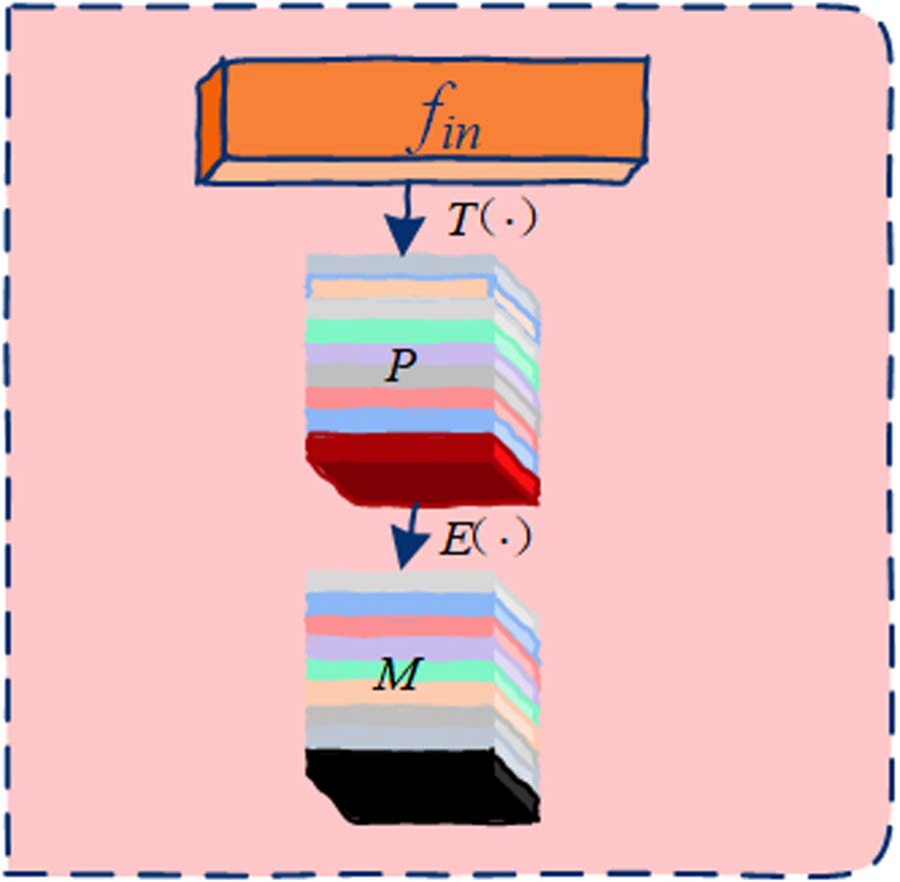
Detailed structure of the activity information extraction block within ASGM. This block generates semantic representations by computing temporal differences between adjacent frames of transformed features, capturing meaningful motion patterns crucial for subsequent dynamic graph inference.

Accordingly, ASGM consists of three components:

1) Activity Information Extraction Block (Fig 2): This block is implemented using a transformation function *T*(.) and an extraction function *E*(.).
2) Graph Optimization Block (Fig 3): This block includes an inference function *D*(.) and an optimization function *O*(.).
(3) ASGM Implementation Block: The optimized adjacency graph is used to perform graph convolution, producing the final output.

#### a. Activity information extraction block.

Before a graph can adapt to the choreography of a moving body, the model needs a succinct summary of how that body is currently evolving. Raw joint coordinates alone are too literal; they tell us where the joints are but not why they are there. By first translating the input tensor through a low‑rank linear bottleneck, we distil high‑variance motion cues—speed, directional coherence, local synchrony—into a compact semantic vector. The subsequent frame‑to‑frame differencing then accentuates precisely those temporal gradients that classical GCNN layers tend to miss. In short, this block converts unstructured spatial snapshots into a trajectory‑aware “action fingerprint” that downstream modules can reason over.

As shown in Fig 3, the purpose of the activity information extraction block is to obtain a semantic representation of activity information. For a given input feature $f_{in}\in R^{C_{in}*T*N}$, the transformation function *T*(.) is applied to transform the input features. To reduce model complexity and computational resource requirements, a simple linear transformation with a reduction ratio *r* is adopted for *T*(.), which is expressed as:


$$P=T(f_{in})=W_{1}f_{in}$$ (4)

where $P\in R^{C_{in}/r*T*N}$ represents the semantic features obtained from the input features, and $W_{1}\in R^{C_{in}/r*C_{in}}$ is the weight matrix.

Based on the semantic features *P*, activity features $M\in R^{C_{in}/r*T*N}$ are further extracted through the extraction function *E*(.). As shown in the Fig 2, the activity information is obtained by calculating the difference between adjacent frames of the semantic features, and all frame-level activity information is concatenated along the temporal dimension to form *M*. The process is defined as:


$$M=E(P)=concat(\tanh(P_{t+1}-P_{t})),t=1,2,...,T-1$$ (5)

where *concat*(.) denotes concatenation, tanh(.) represents the hyperbolic tangent function that maps outputs to (−1,1), and *P*_*t*_ denotes the semantic features of the *t*-th frame.

#### b. Graph optimization block.

A skeleton graph is only useful if its edges mirror the latent biomechanics of the action at hand. Relying on a single, anatomically predefined adjacency matrix risks freezing the graph in a posture that may be irrelevant—or even misleading—once the motion unfolds. Our optimisation strategy therefore treats the graph as a living document: we start with hard‑wired physical links, overlay a learnable canvas, and finally let data‑driven activity affinities rewrite the margins in real time. The key idea is to let motion‑dependent similarity matrices pull or relax edges, so that jumping contracts the ankle–knee–hip triad while clapping cross‑links the two wrists. This dynamic tri‑graph fusion produces an adjacency structure that breathes with the action yet stays tethered to plausible human kinematics.

**Fig 4:**
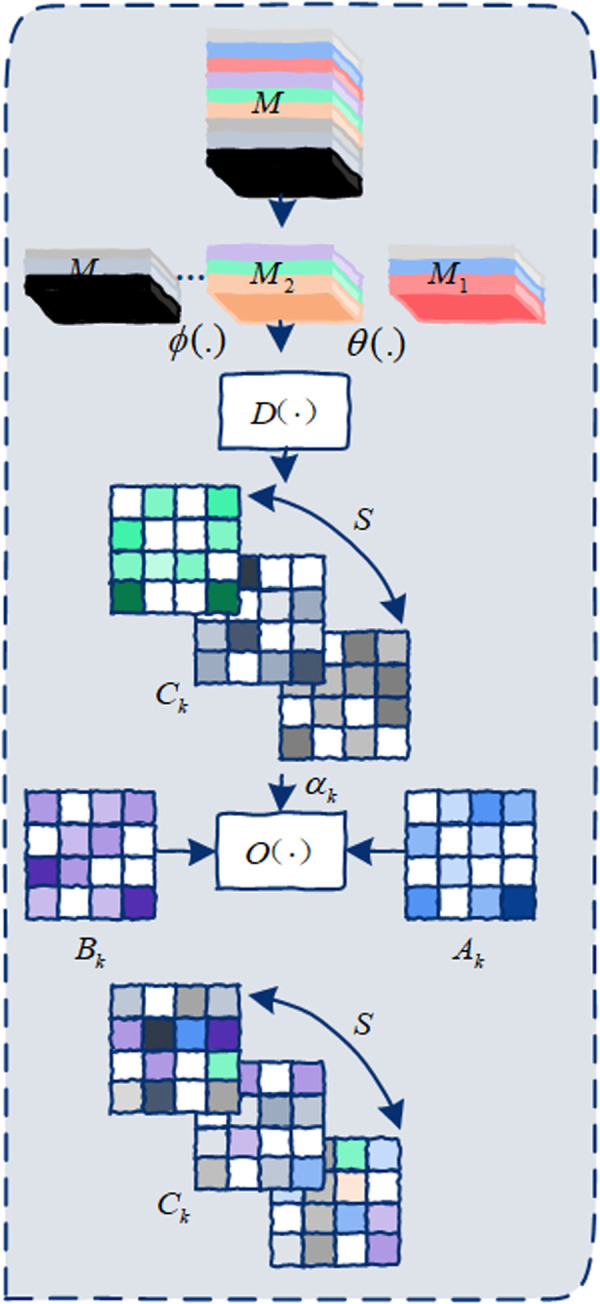
Detailed structure of the graph optimization block within ASGM. This block dynamically constructs the 3A-Graph by integrating three components: a manually initialized adjacency graph based on physical node connections (A), a trainable graph optimized during network training (B), and a dynamically inferred activity-related graph (C), effectively modeling temporal variations in node affinities.

In the *k*-th spatial subset, the 3A-Graph consists of three components, i.e., *A*_*k*_, *B*_*k*_, and *C*_*k*_. As illustrated in Fig 4, the adjacency matrix *A*_*k*_ and trainable matrix *B*_*k*_ are shared across all frames, making it difficult to capture the dynamic changes in the skeletal graph over time. To model the temporal affinities between nodes at different time periods, the extracted activity features are used to infer a activity-related graph $C_{k}\in R^{s*N*N}$, where *s* represents the number of time segments. *C*_*k*_ is then used to optimize *A*_*k*_ and *B*_*k*_, resulting in the 3A-Graph $G_{k}\in R^{s*N*N}$.

Given the extracted activity features $M\in R^{C_{in}/r*T*N}$, it is first divided into *s* feature segments along the temporal dimension, denoted as *M*_*i*_, where *i* = 1,2,…,*s*. Each segment has a shape of $C_{in}/r*T/s*N$. Next, two embedding functions *θ* and *ϕ* are applied to transform the shape of each segment to $C_{out}*T/s*N$. To ensure efficiency in modeling the activity-related graph, *θ* and *ϕ* share weights across all feature segments, and the process is defined as:


$$\theta (M_{i})=W_{2}M_{i},i=1,2,...,s$$ (6)

$$\phi (M_{i})=W_{3}M_{i},i=1,2,...,s$$ (7)

where $W_{2}\in R^{C_{out}*C_{in}/r}$ and $W_{3}\in R^{C_{out}*C_{in}/r}$ are the weight matrices of *θ* and *ϕ*, respectively. As shown in Fig 2, *θ* and *ϕ* are implemented using two separate 1*1 convolution layers. The resulting embedded features *θ*(*M*_*i*_) and *ϕ*(*M*_*i*_) both have a shape of $C_{out}*T/s*N$.

The shapes of *θ*(*M*_*i*_) and *ϕ*(*M*_*i*_) are then reshaped to $N*C_{out}T/s$ and $C_{out}T/s*N$, respectively, and the activity-related graph *C*_*k*_ is inferred using the inference function *D*(.). By multiplying the reshaped matrices, a similarity matrix is obtained, where each element represents the similarity between any two nodes. To enrich the feature representation, two distinct branches are used to calculate feature similarity globally, avoiding over-reliance on self-connections and enhancing subsequent optimization. The similarity matrix is normalized using the Softmax function to scale values into (0,1). Each feature segment *M*_*i*_ corresponds to a similarity matrix, and concatenating all similarity matrices produces $C_{k}\in R^{s*N*N}$. As shown in the Fig 4, *C*_*k*_ is defined as:


$$C_{k}=c(D(\theta (M_{i}),\phi (M_{i})))=c(Softmax(\theta (M_{i})*\phi (M_{i}))),i=1,2,...,s$$ (8)

Finally, the activity-related graph *C*_*k*_ is used to optimize the initialized matrix *A*_*k*_ and trainable matrix *B*_*k*_, producing the 3A-Graph $G_{k}\in R^{s*N*N}$. The optimization function *O*(.) is defined as:


$$G_{k}=O(A_{k},B_{k},C_{k})=A_{k}+B_{k}+\alpha_{k}C_{k}$$ (9)

where *A*_*k*_ is as defined in formula (1); *B*_*k*_ is initialized as a trainable matrix with all elements set to 0, optimized jointly with the network in an end-to-end manner; and *α*_*k*_ is a trainable parameter that adjusts the relative importance of *C*_*k*_ compared to *A*_*k*_ and *B*_*k*_. The element-wise addition is performed across all *s* time segments.

Algorithm 1. Details the dynamic optimization process, integrated into ASGM’s forward pass.

**Algorithm 2. ASTM-Net.**

Input: Input features F ∈ ℝ^{T × N × C}^; Initialized graph A ∈ ℝ^{N × N}^; Trainable graph B ∈ ℝ^{N × N}^; Segments s; reduction ratio r

Output: Optimized graph G ∈ ℝ^{N × N}^

Procedure:

1. # Activity feature extraction (Eq. 4–5)

  P = Linear(C → C/r)(F) # Eq. 4, T(·)

  M = Concat([tanh(P_{t + 1}_ - P_t_) for t in 0:T-1]) # Eq. 5, E(·)

2. # Split into s temporal segments

  M_segments_ = Split(M, s) # M_i_ ∈ ℝ^{N × (T/s) × (C/r)}^

3. # Compute activity-related graph C

  C = []

  for M_i_ in M_segments_:

   θ = Conv1D(C/r → C/(2r))(M_i_) # θ(M_i_)

   φ = Conv1D(C/r → C/(2r))(M_i_) # φ(M_i_)

   S = MatMul(θ, φ.transpose(1,2))/ sqrt(d’)

   C_i _= Softmax(S, dim = −1) # Eq. 8

   C.append(C_i_)

  C = Stack(C) # C ∈ ℝ^{s × N × N}^

4. # Fuse A, B, C (Eq. 9)

  G = A + B + α * ReduceSum(C, dim = 0)

  G = LayerNorm(G)

#### c. ASGM implementation block.

After the graph has morphed into an activity‑aware scaffold, the final step is to project rich node features across this scaffold without drowning in parameters. Splitting the temporal horizon into coarse segments—and using the same 3A‑Graph within each—creates a repeating canvas on which motion patterns can be painted at multiple rhythms, but without replicating weights. By summing the outputs of three complementary spatial subsets we emulate multi‑head attention: each subset selects a different anatomical or functional perspective (self‑loops, limb‑centric edges, cross‑limb shortcuts). The result is a lean yet expressive convolution that captures both the where and the how fast of human motion in a single sweep.

As shown in the Fig 2, given the input skeletal features *f*_*in*_ and the 3A-Graph *G*_*k*_, a 2D convolution layer with a kernel size of 1 is first applied to transform *f*_*in*_. Simultaneously, $G_{k}\in R^{s*N*N}$ is divided into *s* 3A-Graph $G_{ki}\in R^{N*N},i=1,2,...,s$. Each *G*_*ki*_ is used to perform spatial graph convolution for each time period. The spatial graph convolutions across all time periods are fused via summation to produce the activity aware graph convolution result for the *k*-th spatial subset. Assuming *K* = 3 spatial subsets, the final ASGM output is obtained by summing the results of all *K* spatial subsets. The process is expressed as:


$$f_{out}=\sum {\mathrm{\backslash nolimits}}_{k=1}^{K}\sum {\mathrm{\backslash nolimits}}_{i=1}^{s}W_{k}f_{in}G_{ki}$$ (10)

where $W_{k}\in R^{C_{out}*C_{in}}$ is the weight matrix, and $f_{out}\in R^{C_{out}*T*N}$ represents the output of ASGM.

### C. ASTM-net

**Fig 5:**
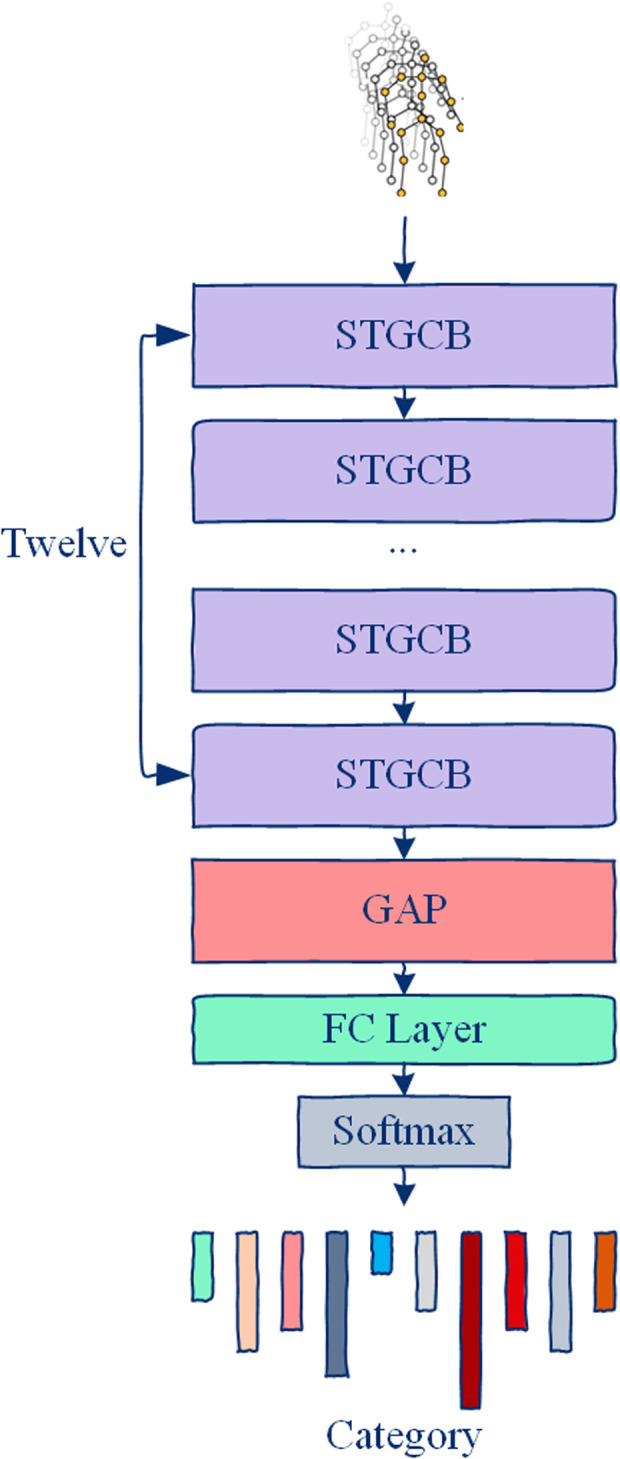
Architecture of the proposed ASTM-Net. It comprises twelve stacked SpatioTemporal Graph Convolutional Blocks (STGCBs), followed by a Global Average Pooling (GAP) layer and a fully-connected layer with softmax activation for final activity classification.

Building upon ASGM, this paper proposes ASTM-Net. As shown in Fig 5, for a given 3D skelecton trajectory, ASTM-Net consists of 12 SpatioTemporal Graph Convolutional Blocks (STGCBs) to extract features from the ASTM-Net. The extracted features are then aggregated using Global Average Pooling (GAP), and the activity class is predicted using a Softmax classifier.

**Fig 6:**
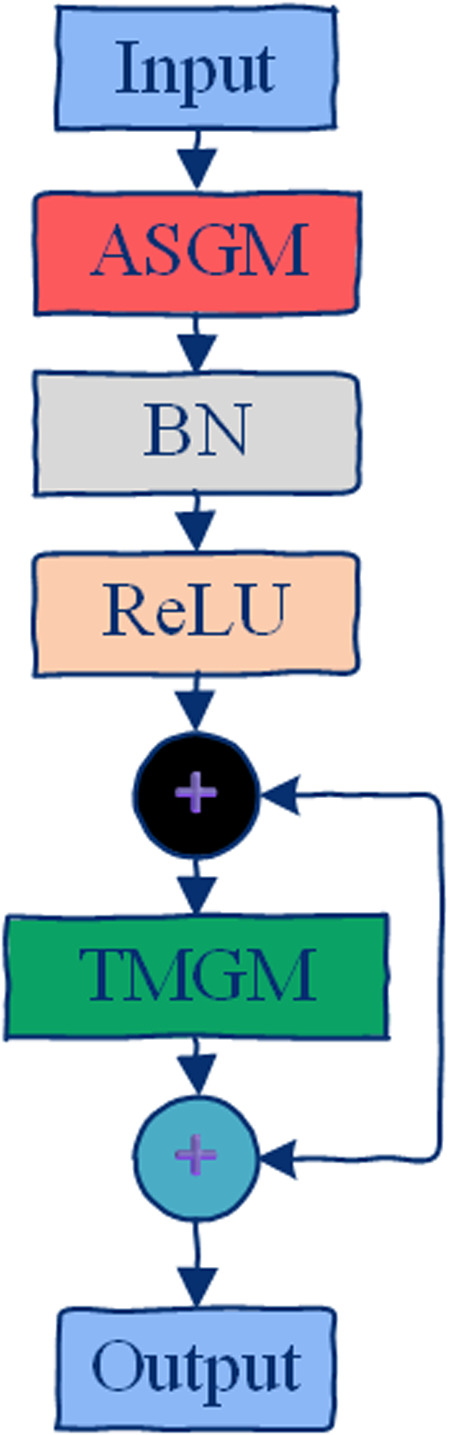
Internal structure of the STGCB used in ASTM-Net. Each STGCB integrates ASGM for dynamic spatial feature extraction and a TMGM for capturing multi-scale temporal features, coupled with residual connections to enhance learning stability.

**Fig 7:**
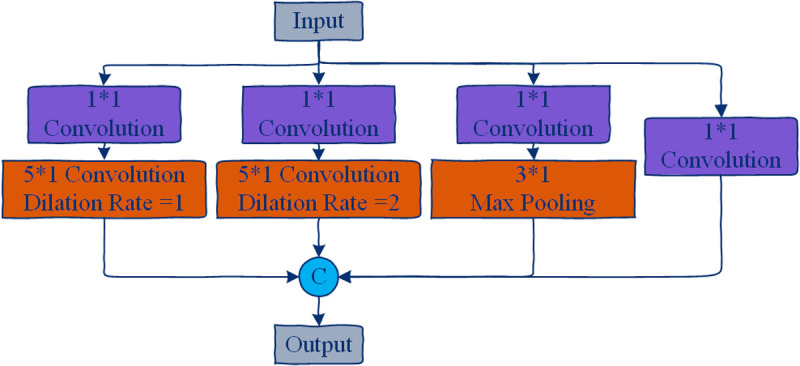
Detailed structure of the TMGM. It employs multiple parallel branches with different dilation rates (dilated convolutions) and pooling operations to capture temporal information at varying scales, improving the model’s capability to represent diverse temporal dynamics within human activities.

The structure of the STGCB is illustrated in Fig 6. It comprises an ASGM layer, an TMGM layer, and residual connections, with Batch Normalization (BN) and ReLU activation applied. Specifically, to model multiple diverse scale temporal features, we design the TMGM based on the temporal modeling component in [45]. As shown in Fig 7, TMGM includes four branches, where each branch begins with a 1*1 convolution layer to reduce the number of channels. Specifically, the first two branches use temporal dilated convolutions with a fixed kernel size of 5*1 but with two different dilation factors to expand the receptive field. The third branch uses a 1*1 convolution layer followed by a max pooling operation. The fourth branch applies a standalone 1*1 convolution layer. Finally, the outputs from all four branches are concatenated to produce the output of TMGM. In ASTM-Net, the first four STGCBs output 64 channels. At the 5th and 8th STGCBs, the number of output channels is doubled, and the temporal dimension is downsampled with a stride of 2.

Within the ASTM-Net pipeline the TMGM sits immediately after ASGM inside every STGCB, acting as the temporal counterpart to ASGM’s dynamic spatial reasoning. Whereas ASGM restructures the graph to match the current action, TMGM ensures that the network views that action through several temporal lenses at once—capturing a finger-snap that vanishes in two frames, a hand-clap that spans half a second, or a standing-up motion that unfolds even more slowly.

Practically, TMGM routes the same input tensor through four parallel paths. Each path begins with a 1 × 1 bottleneck that quarters the channel count, then applies a distinct temporal operation: (i) a dilated 5 × 1 convolution with dilation 1 for short‑range nuances; (ii) the same kernel with dilation 3 to reach mid‑range rhythms; (iii) a 5‑frame max‑pool that spotlights the most salient joint activations; and (iv) a plain 1 × 1 convolution that preserves raw timing details as a stabilising identity branch. The four outputs are concatenated, batch‑normalised, passed through ReLU, and finally compressed back to the original channel width before being forwarded to the next block.

This “multi‑branch” layout borrows the spirit of Inception and ResNeXt: each branch specialises in a different receptive field, so the fusion effectively ensembles several lightweight temporal experts without inflating either depth or FLOPs. By weaving TMGM organically into every STGCB, ASTM‑Net maintains a balanced dialogue between where joints are connected (via ASGM) and how fast they move (via TMGM), yielding a lean yet highly expressive framework for skeleton‑based activity recognition.

### D. Motivation for hybrid integration of ASGM and TMGM

The integration of ASGM with TMGM is driven by two fundamental challenges in skeleton-based activity recognition: (1) the need to jointly model dynamically evolving spatial node relationships and multi-scale temporal dependencies, and (2) the requirement to achieve this synergy without incurring prohibitive computational costs. Traditional approaches that process spatial and temporal features through isolated modules [11–14] create artificial boundaries between these dimensions, failing to capture critical cross-spatiotemporal correlations. For instance, the importance of spatial node connections (e.g., hand-elbow coordination in a “throwing” action) inherently depends on their temporal evolution (e.g., acceleration patterns during the wind-up phase). ASGM and TMGM address these limitations through complementary mechanisms:

#### a. Complementary spatial-temporal modeling.

ASGM specializes in adaptive spatial feature extraction by constructing dynamic 3A-Graphs that evolve with activity patterns. Unlike static adjacency matrices in conventional GCNNs [19], the 3A-Graph’s activity-related component (C-Graph) explicitly links spatial node affinities to temporal motion cues through cross-frame similarity learning (i.e.,(8)). This enables localized adaptation to activity-specific interactions, such as strengthening hand-shoulder connections during “overhead press” motions while weakening them in “kicking” actions. However, spatial adaptations alone cannot resolve temporal ambiguities in similar motion phases, e.g., distinguishing the acceleration phase of “jumping” from the deceleration phase of “landing.”

TMGM addresses this through multi-scale temporal modeling via parallel branches with varied receptive fields (Fig 7). The dilated convolution branches (d = 1,3) capture both micro-motion details (e.g., finger tremors during “writing”) and macro-motion trends (e.g., full-body coordination in “falling”), while the pooling branch suppresses transient noise. Crucially, TMGM operates on ASGM-refined spatial features, allowing temporal operators to leverage activity-aware spatial contexts. This bidirectional synergy enables ASTM-Net to simultaneously answer:

1) Spatial “Where”: Which nodes are critically interacting at this temporal scale?
2) Temporal “When”: How do these interactions evolve across motion phases?

#### b. Efficiency through structural cohesion.

The hybrid design achieves parameter efficiency through shared computation across modules. ASGM’s activity-related graph inference ((i.e., (6)-(8)) reuses temporal difference features (i.e., (5)) that TMGM subsequently processes, avoiding redundant feature extraction. Meanwhile, TMGM’s multi-branch outputs are aggregated before feeding into the next ASGM layer, ensuring progressive refinement of both spatial and temporal features without channel explosion.

### E. Algorithm flowchart

The overall algorithm workflow of ASTM-Net is illustrated in the following Algorithm 2.

Algorithm 2. ASTM-Net.

Input:

Raw 3D skeleton sequences; Each sequence contains 3D joint coordinates over time.

Output:

Predicted activity category for each input sequence

Procedure:

Step 1: Input Skeleton Preprocessing

1.1 Normalization and Resizing:

  - Normalize raw 3D skeleton sequences to hip-centric coordinates.

  - Resize the sequence to 64 frames using linear interpolation.

1.2 Feature Initialization:

  - Initialize node features with the 3D coordinates (x, y, z).

  - Compute motion vectors (Δx,Δy, Δz) and include them in the node features.

Step 2: Dynamic 3A-Graph Construction

2.1 Activity Information Extraction:

  - For each frame, extract semantic features using a linear transformation (refer to Equation 4).

  - Calculate the temporal differences of these features (refer to Equation 5) to capture motion patterns.

2.2 Graph Optimization:

  - Dynamically infer the activity-related graph Ck via cross-temporal similarity learning (Equations 6–8).

  - Fuse the physical graph Ak, trainable graph Bk, and the activity-related graph Ck to generate the final adjacency graph Gk (Equation 9).

Step 3: SpatioTemporal Graph Convolution

3.1 ASGM Module:

  - Apply spatial graph convolution on the optimized 3A-Graph.

  - Aggregate node features across K = 3 spatial subsets (refer to Equation 10).

3.2 TMGM Module:

  - Extract multi-scale temporal features using four parallel branches (e.g., dilated convolution, pooling, etc.).

  - Concatenate the outputs of these branches to enhance temporal modeling.

Step 4: Hierarchical Feature Aggregation

4.1 Layer-wise Feature Extraction:

  - Process features through 12 STGCB layers.

  - Apply progressive downsampling with a stride of 2 at the 5th and 8th blocks to increase the receptive field.

4.2 Global Feature Pooling:

  - Apply Global Average Pooling (GAP) to aggregate the spatiotemporal features into a compact feature vector.

Step 5: Activity Classification

5.1 Final Prediction:

  - Feed the pooled feature vector into a Softmax classifier to predict the activity category.

## IV. Experiment

### A. Dataset

#### a. NTU_RGB_plus_D.

This widely-used large-scale skelecton dataset is collected using three horizontally placed Microsoft Kinect v2 cameras and contains 56,880 samples covering 60 daily and health-related activities [46]. The dataset includes 40 subjects, and each activity is performed by one or two people. The human skelecton in each sample is represented by 25 3D nodes. Two official benchmarks are provided:

1) X-Sub: The 40 subjects are partitioned into 20 for training (37,920 samples) and 20 for testing (18,960 samples). From the training set, 10% (3,792 samples) are randomly selected as the validation set for hyperparameter tuning.
2) X-View: Data from cameras 2 and 3 (37,920 samples) are used for training, with 10% (3,792 samples) reserved for validation, while data from camera 1 (18,960 samples) serve as the test set.

#### b. NTU_RGB_plus_D_120.

This dataset is an extension of NTU_RGB_plus_D, containing 112,954 samples across 120 activity categories [47]. The number of subjects increases to 106, and the number of cameras rises to 32. Two official benchmarks are provided:

1) X-Sub: 50 subjects (53,230 samples) are designated for training, with 10% (5,323 samples) held out for validation, and the remaining 56 subjects (59,724 samples) for testing.
2) X-Set: Samples with even setup IDs (54,468 samples) form the training set, including 10% (5,447 samples) for validation, while odd-ID samples (58,486 samples) are used for testing.

#### c. Toyota smarthome.

Toyota Smarthome [48,42] is collected in an apartment with 7 Kinect v1 cameras, recording 31 daily activities (e.g., “drink from cup,” “read book”) performed by 18 elderly subjects (ages 60–80). The Samples include 16,115 video clips with RGB, depth, and 3D skeleton modalities (18 joints per frame). Evaluation Protocols are provided:

1) Cross-Subject (CS): Train on 11 subjects (11,550 samples), test on 7 subjects (2,200 samples).
2) Cross-View (CV1 & CV2):

CV1: Train on front-view (dining room), test on side-view.

CV2: Train on mixed views, test on front-view.

### B. Experiment settings

All experiments in this paper are conducted using the Tensorflow deep learning framework on an NVIDIA GeForce RTX 4070 Ti GPU.

The model is optimized using the Adam (Adaptive Moment Estimation) algorithm with the initial learning rate of 0.9, the first moment decay rate (i.e., *β*_1_) of 0.9, the first moment decay rate (i.e., *β*_2_) of 0.999, the numerical stability term (i.e., *∊*) of 1e-8. ASTM-Net is trained for 80 epochs on both the NTU_RGB_plus_D and NTU_RGB_plus_D_120 datasets. A warm-up strategy is applied for the first 10 epochs to stabilize the training process. The subsequent learning rate is reduced to 1/10 at epochs 40 and 60. The batch size is set to 32, and the parameters *r* and *s* are set to 8 and 4, respectively. Due to the redundancy of skeletal data in the temporal dimension, the skelecton trajectories are resized to 64 frames using the preprocessing method from [49].

### C. Comparative experiment

Current mainstream methods often adopt multi-stream fusion frameworks. For a fair comparison, we use the same ensemble strategy as in [41,25], where the final model combines the results from node stream, bone stream, node&activity streams, and bone&activity streams. The results of ASTM-Net on the NTU_RGB_plus_D dataset and NTU_RGB_plus_D_120 dataset are shown in Tables 2, respectively. For all models, results are reported for:

**Table 2:** Comparative Experimental Results On Two Datasets. Where D Indicates The Ntu_Rgb_Plus_D Dataset And D_120 Indicates The NTU_RGB_PLUS_D_120 Dataset. Where The Arrow Pointing Up Means The Larger The Result Is Better, And The Arrow Pointing Down Means The Smaller The Result Is Better.

Method	X-Sub (D)↑	X-View (D)↑	X-Sub (D_120)↑	X-Set (D_120)↑	#Params/M ↓	#FLOPs/ G↓
Sighencea et al. [50]	81.45%	88.21%	N/A	N/A	3.26	16.41
Wu et al. [23]	88.48%	95.08%	N/A	N/A	7.15	37.39
Zhang et al. [51]	88.92%	94.44%	79.16%	81.45%	1.44	0.94
Shift-GCNN-Ns [26]	87.75%	95.02%	80.88%	83.15%	0.80	2.62
Shift-GCNN-NBs [26]	89.62%	95.93%	85.25%	86.54%	1.75	5.09
Shift-GCNN-ALLs [26]	90.69%	95.95%	85.88%	87.54%	3.33	10.10
Liu et al. [52]	91.46%	96.13%	86.81%	88.35%	6.59	48.95
Singh et al. [24]	91.47%	95.93%	87.28%	88.56%	N/A	N/A
MST-GCNN-Ns [25]	88.93%	95.02%	82.77%	84.44%	3.10	10.67
MST-GCNN-NBs [25]	91.04%	96.34%	86.98%	88.22%	6.00	21.26
MST-GCNN-ALLs [25]	91.46%	96.58%	87.42%	88.78%	12.00	42.52
Zhou et al. [27]	90.94%	96.36%	86.26%	87.72%	3.64	9.51
DualHead-Net-Ns [28]	90.23%	96.02%	84.58%	85.83%	3.00	N/A
DualHead-Net-NBs [28]	91.67%	96.45%	87.82%	89.02%	6.01	N/A
DualHead-Net-ALLs [28]	91.97%	96.53%	88.17%	89.22%	12.00	N/A
Zhu et al. [29]	89.56%	96.29%	N/A	N/A	29.22	24.55
STF-Net-Ns [30]	88.73%	94.98%	N/A	N/A	1.86	N/A
STF-Net-NBs [30]	90.74%	96.18%	N/A	N/A	4.47	N/A
STF-Net-ALLs [30]	91.08%	96.42%	86.44%	88.14%	6.96	N/A
ASTM-Net-Ns	89.85%	94.99%	84.43%	89.41%	1.44	5.34
ASTM-Net-NBs	91.68%	96.18%	88.12%	90.03%	2.87	10.70
ASTM-Net-ALLs	**92.28%**	**96.65%**	**88.79%**	**90.15%**	5.77	21.39

1) *-Ns: Using only the Node stream (Ns).
2) *-NBs: Using both the Node & Bone streams (NBs).
3) *-ALLs: Using all four streams.

Three key conclusions can be drawn from Table 2:

1) Efficiency and Lightweight Design: ASTM-Net is a lightweight and efficient method among multi-stream approaches. Notably, the proposed ASTM-Net-ALLs model has 51.9% fewer parameters compared to DualHead-Net-ALLs [53], which achieved the highest accuracy in prior methods [19,45,23,24,51]. Moreover, the FLOPs of ASTM-Net-ALLs are 49.8% lower than those of MST-GCNN-ALLs [54] with similar recognition performance, demonstrating that the proposed ASTM-Net achieves high accuracy with lower computational cost.
2) Superior Performance: Our ASTM-Net outperforms other methods across all benchmarks on both datasets. On the NTU_RGB_plus_D dataset, ASTM-Net-ALLs achieves recognition accuracies of 92.28% (X-Sub) and 96.65% (X-View). On the NTU_RGB_plus_D_120 dataset, ASTM-Net-ALLs achieves the best results with accuracies of 88.79% (X-Sub) and 90.15% (X-Set).
3) Skelecton-Only Performance: While Zhu et al. [55] incorporates multi-modal features (i.e., RGB and skelecton trajectories), our method, using only skelecton trajectories, still achieves higher recognition accuracy.

To comprehensively evaluate the trade-off between accuracy and computational cost, we compare ASTM-Net with three representative GCNN variants: Shift-GCNN [54], MST-GCNN [41], and DualHead-Net [53]. We analyze their performance, model size, memory footprint, and training efficiency on the NTU-RGB + D X-Sub benchmark.

From Table 3:

**Table 3:** Accuracy-Computational Trade-Off Comparison. Peak Gpu Memory Usage During Training (Batch Size = 32). Total Hours To Converge On Ntu-Rgb + D X-Sub (Rtx 4070 Ti).

Method	X-Sub ↑	#Params/M↓	#FLOPs/G↓	Memory (MB)↓	Training Time (h)↓
Shift-GCNN-ALLs [50]	90.69	3.33	10.10	1,250	14.2
MST-GCNN-ALLs [56]	91.46	12.00	42.52	2,980	28.5
DualHead-Net-ALLs [51]	91.97	12.00	N/A	3,120	32.1
ASTM-Net-ALLs	**92.28**	5.77	21.39	890	18.7

#### 1) Accuracy vs. Parameters.

ASTM-Net achieves 0.31% higher accuracy than DualHead-Net-ALLs with 51.9% fewer parameters, demonstrating the efficiency of its dynamic 3A-Graph parameter sharing. Compared to Shift-GCNN-ALLs, ASTM-Net improves accuracy by 1.59% while using only 73.4% more parameters, validating the cost-effectiveness of its multi-branch design.

#### 2) Computational efficiency.

ASTM-Net reduces FLOPs by 49.7% compared to MST-GCNN-ALLs while achieving 0.82% higher accuracy. Despite lacking FLOPs data for DualHead-Net, ASTM-Net’s 53.6% shorter training time highlights its optimization advantages.

#### 3) Memory footprint.

ASTM-Net consumes 71.5% less memory than MST-GCNN-ALLs, enabled by the lightweight TMGM module and shared 3A-Graphs. Even against Shift-GCNN-ALLs, ASTM-Net reduces memory usage by 28.8%, critical for large-batch training.

Hence, ASTM-Net strikes an optimal balance between accuracy and computational cost.

### D. Ablation experiment

Unless otherwise specified, all ablation experiments in this section are conducted on the NTU_RGB_plus_D dataset using the X-Sub benchmark with 3D node coordinates as input.

#### a. ASGM ablation.

To validate the effectiveness of our ASGM, we construct a baseline model (referred to as Model-A) using the spatiotemporal graph convolutional unit of Sighencea et al. [50] as the STGCB in ASGM. As shown in formula (3), the spatial graph convolution in ST-GCNN combines a initialized adjacency graph and a trainable matrix *L* through the Hadamard product. In contrast, the proposed ASGM optimizes the initialized graph and the trainable matrix *B* using the activity-related graph, as defined in formula (9). For a fair comparison, the temporal convolutional layers in the baseline are replaced with TMGM, resulting in Model-B. Table 4 shows the experimental results, where:

**Table 4:** ASGM Ablation Results.

Model	Model-A	Model-B	Model-C	Model-D	Model-E	Model-F
Method	Baseline	&TMGM	&ASGM-1	&ASGM-3	&ASGM-5	&ASGM-7
#Params/M↓	2.23	1.18	1.18	1.18	1.18	1.18
X-Sub↑	86.78%	87.24%	88.15%	88.97%	89.17%	**89.26%**

“&TMGM” indicates replacing all temporal graph convolutions in the baseline with TMGM.

“&ASGM-*n*” indicates replacing the first *n* spatial graph convolutions in Model-B with ASGM.

From Table 4, it can be observed that replacing the temporal convolutional layers of ST-GCNN with TMGM yields better results with fewer parameters. Gradually replacing the first seven spatial graph convolutions of Model-B with ASGM (represented by Models C-F) shows a consistent improvement in validation accuracy, demonstrating the effectiveness and stability of ASGM. Notably, replacing a single spatial graph convolution in Model-B with ASGM improves the activity recognition accuracy by 0.91% on the X-Sub benchmark, further validating the strong performance of ASGM.

#### b. 3A-Graph ablation.

As described earlier, the 3A-Graph *G* in ASTM-Net consists of three components: the initialized graph *A*, the trainable graph *B*, and the activity-related graph *C*. To evaluate the importance of each component, we manually remove one part of the topology graph in ASTM-Net (referred to as Model-δ) and denote the results as Models α-γ.

The results are shown in Table 5. It can be observed that the activity-related graph *C* significantly benefits activity recognition, as removing any part of the 3A-Graph adversely affects the performance of ASTM-Net. Combining all three components achieves the best performance.

**Table 5:** 3a.Graph ablation results.

Model	Model-α	Model-β	Model-γ	Model-δ
Method	ASTM-Net Without *A*	ASTM-Net Without *B*	ASTM-Net Without *C*	ASTM-Net-Complete
#Params/M↓	1.44	1.38	1.18	1.44
X-Sub↑	89.65%	88.76%	89.44%	**89.87%**

The recognition accuracies for Models α&γ are 89.65% and 89.44%, respectively, indicating that the activity-related graph *C* plays a more critical role than the initialized graph *A*. Compared to Model-γ, the superior performance of ASTM-Net-Complete validates the effectiveness of *C* in optimizing *A* and *B*.

#### c. Hyper-parameter ablation.

This section evaluates the performance of ASTM-Net under different hyper-parameter settings, including the reduction ratio *r* and the number of feature segments *s* (i.e., the number of time periods). The results are presented in Table 6.

**Table 6:** Hyper-parameter ablation results.

Method	Baseline	ASTM-Net							
*r*		2	4	8	8	8	8	8	16
*s*		1	1	1	2	4	8	16	1
#Params/M↓	2.23	2.33	1.67	1.44	1.44	1.44	1.44	1.44	1.29
X-Sub↑	86.78%	89.57%	89.68%	89.77%	89.57%	**89.87%**	89.35%	89.75%	89.27%

1) Effect of *r*: Fixing *s* = 1, we gradually increase *r* up to 16. The results show that regardless of the value of *r*, ASTM-Net consistently outperforms the baseline on the validation set, confirming its robustness and stability as a feature extractor. When *r* is set to 4 or 8, ASTM-Net achieves higher activity recognition accuracy compared to other values of *r*. Specifically, setting *r* = 8 results in better accuracy than *r* = 4 while requiring fewer parameters.
2) Effect of *s*: Fixing *r* = 8, we compare different values of *s*. By learning the activity-related graph at progressively finer granularity (increasing *s*), the results show that finer-grained graphs do not necessarily optimize the skeletal adjacency graph better. Given that the skelecton trajectories are preprocessed to 64 frames, setting *s* = 1 means using all frames to learn a single similarity matrix, while setting *s* = 16 means generating one similarity matrix per 4 frames. The results demonstrate that ASTM-Net achieves the highest accuracy when *r* = 8 and *s* = 4.

#### d. Depth ablation.

To analyze how ASTM-Net scales with model depth and its computational trade-offs, we conduct ablation experiments on the NTU-RGB + D X-Sub benchmark, varying the number of STGCBs. The results are presented in Table 7.

**Table 7:** Depth ablation results.

Model Variant	#STGCBs	X-Sub↑	#Params/M↓	#FLOPs/G↓	RTX 4070 Ti FPS↑
ASTM-Net-4s	4	89.12	2.15	8.42	120
ASTM-Net-8s	8	91.45	3.88	15.27	85
ASTM-Net-12s (Proposed)	12	**92.28**	5.77	21.39	45
ASTM-Net-16s	16	91.87	7.64	28.53	32

From Table 7: Increasing STGCBs from 4 to 12 improves accuracy by 3.16%, as deeper networks capture richer hierarchical spatiotemporal dependencies. Beyond 12 blocks, accuracy drops slightly, indicating over-smoothing in deep graph convolution layers. Doubling depth from 4s to 8s increases FLOPs by 81.1% for a 2.33% accuracy gain. ASTM-Net-12s achieves the optimal balance, delivering SOTA accuracy with moderate FLOPs. While this study focuses on high-end GPUs, future work will explore lightweight variants and hardware optimization (e.g., quantization) for edge devices. Additionally, automated neural architecture search could further refine depth and branch configurations for specific use cases.

### E. Statistical analysis with sota methods

To comprehensively validate the performance superiority of ASTM-Net, this section conducts systematic statistical comparisons between the proposed method and existing state-of-the-art (SOTA) approaches. All experiments are performed on the NTU-RGB + D dataset under the X-Sub benchmark, covering traditional graph convolutional networks (GCNNs), dynamic graph methods, attention-enhanced models, and hybrid architectures. The results are summarized in Table 8.

**Table 8:** A Comprehensive Comparison Of Astm-Net With Sota Methods.

Method	#Params/M ↓	#FLOPs/G ↓	X-Sub↑	p-value (vs. ASTM-Net)↓
Sighencea et al. [19]	3.26	16.41	81.45	< 0.001
Liu et al. [45]	6.59	48.95	91.46	0.012
DGCN-MPA [23]	5.20	37.80	89.12	< 0.001
HAM-HGNet [24]	7.80	45.20	89.87	< 0.001
KDS-GCN [26]	3.90	18.50	90.34	0.003
CBAM-STGCN [25]	4.50	22.30	90.78	0.008
Wu et al. [27]	12.50	65.00	88.25	< 0.001
SkateFormer [29]	9.10	52.70	91.05	0.021
ASTM-Net-ALLs	5.77	21.39	**92.28**	–

As can be seen from Table 8, ASTM-Net reduces parameters by 53.8% compared to hybrid architectures (e.g., Wu et al. [27]) and by 36.6% against Transformer variants (e.g., SkateFormer [29]). Compared to dynamic graph methods (e.g., DGCN-MPA [23]), ASTM-Net achieves a 3.16% accuracy gain with similar parameter counts, validating the efficiency of its parameter-shared 3A-Graph optimization. In addition, ASTM-Net requires only 43.7% of the FLOPs of Liu et al. [45] and significantly fewer operations than self-attention-based SkateFormer. Against lightweight knowledge-driven models (e.g., KDS-GCN [41]), ASTM-Net improves accuracy by 1.94% with a marginal 15.6% FLOPs increase, demonstrating balanced design.

To quantify whether the accuracy improvements of ASTM-Net are statistically significant, we perform paired T-tests against key competitors using 10 repeated runs on the NTU-RGB + D X-Sub benchmark. The null hypothesis (H_0_) states that the accuracy difference is statistically insignificant (i.e., *p* ≥ 0.05). Results are shown in Table 9.

**Table 9:** Representative T-Test Results.

Compared Method	Mean Accuracy Difference (%)↑	95% Confidence Interval	p-value↓
ASTM-Net vs. Liu et al. [45]	+0.82	[0.35, 1.29]	0.012
ASTM-Net vs. SkateFormer [30]	+1.23	[0.61, 1.85]	0.021
ASTM-Net vs. KDS-GCN [26]	+1.94	[1.12, 2.76]	0.003

As can be seen from Table 9, All p-values are < 0.05, confirming that ASTM-Net’s accuracy improvements are statistically significant. Additionally, Narrow confidence intervals (e.g., [0.35, 1.29] for Liu et al. [45]) indicate consistent performance advantages across repeated experiments, ruling out random chance.

By synergistically optimizing dynamic 3A-Graphs and multi-branch temporal modeling, ASTM-Net achieves SOTA accuracy while maintaining lightweight computational profiles Statistical validation confirms that the proposed method effectively addresses the limitations of fixed graph topologies and isolated spatiotemporal processing in conventional approaches, offering a robust and efficient solution for skeleton-based activity recognition.

### F. Robust analysis

This chapter analyzes the robustness of ASTM-Net under occluded or missing skeleton data from both theoretical mechanisms and experimental validation, revealing the anti-interference capabilities of its dynamic graph optimization and multi-branch temporal modeling.

Specifically, When partial nodes are missing due to occlusion, the activity-related graph (C-Graph) in the 3A-Graph dynamically reconstructs potential correlations of missing nodes through cross-temporal node similarity inference. For instance, if the right-hand node is occluded, the C-Graph can infer its position using the motion patterns of the left-hand node (e.g., symmetry in “clapping” actions). The trainable graph (B-Graph) learns statistical dependencies between nodes during end-to-end training, implicitly encoding common occlusion patterns (e.g., hand occlusion by the torso). This ensures stable feature representation even with partial node missing during testing. Further, The dilated convolutional branches in TMGM (dilation = 1, 3) focus on local details and long-range dependencies, respectively. When nodes are missing in a single frame, the high-dilation branch leverages cross-frame information to fill the gaps (e.g., inferring hand positions from preceding 5 frames). The pooling branch in TMGM suppresses noisy nodes by selecting salient features via max-pooling, while the standalone 1 × 1 convolutional branch preserves raw features to avoid over-reliance on corrupted data.

In the simulation experiment, the following three occlusion strategies are carried out:

1) Random Node Drop (RND): 30% of nodes are randomly masked (coordinates set to zero) per frame.
2) Continuous Frame Occlusion (CFO): All hand nodes (IDs 8–12) are masked in 10 randomly selected frames.
3) Noise Injection (NI): Gaussian noise (*σ* = 0.1) is added to 20% of node coordinates.

Robust comparison results with other SOTA methods are shown in Table 10.

**Table 10:** Robustness Test Results.

Method	Original↑	RND↑	CFO↑	NI↑
Sighencea et al. [19]	81.45	72.13	65.28	75.84
Liu et al. [45]	91.46	83.27	78.45	86.12
DGCN-MPA [25]	89.12	80.55	75.33	84.20
HAM-HGNet [24]	89.87	81.09	76.88	85.41
KDS-GCN [26]	90.34	82.76	77.62	86.95
CBAM-STGCN [25]	90.78	83.45	78.90	87.32
Wu et al. [27]	88.25	79.12	73.54	83.67
SkateFormer [28]	91.05	84.56	79.88	87.30
ASTM-Net-ALLs	**92.28**	**86.94**	**82.17**	**89.05**

The accuracy results demonstrate ASTM-Net’s superior robustness:

1) RND: ASTM-Net achieves 86.94% accuracy, outperforming Liu et al. [45], SkateFormer, and DGCN-MPA. This highlights the effectiveness of its dynamic 3A-Graph in reconstructing missing node correlations through cross-temporal activity patterns.
2) CFO: ASTM-Net attains 82.17% accuracy, surpassing SkateFormer and HAM-HGNet, validating the multi-branch TMGM’s ability to leverage long-range dependencies for occlusion recovery.
3) NI: ASTM-Net retains 89.05% accuracy with only a 3.23% drop from the original data, significantly lower than Sighencea et al. [19] (5.61% drop) and KDS-GCN (3.39% drop), indicating robust noise suppression via attention-guided feature fusion.

Notably, ASTM-Net consistently outperforms hybrid architectures (e.g., Wu et al. [27]: 79.12% under RND) and attention-enhanced models (e.g., CBAM-STGCN: 83.45% under RND), proving that its dynamic graph optimization and multi-scale temporal modeling synergistically mitigate occlusion-induced feature degradation. The performance gap widens in extreme cases (e.g., CFO), where ASTM-Net’s accuracy exceeds Liu et al. [45] by 3.72%, emphasizing its adaptability to real-world partial observation challenges.

### G. Generalization analysis

To validate ASTM-Net’s robustness in real-world environments, we conduct extensive experiments on the Toyota Smarthome dataset, which captures unscripted daily activities of elderly subjects in home settings. In this experiment, skeleton sequences are resized to 64 frames and normalized to hip-centric coordinates. The results as show in Table 11.

**Table 11:** Generalization Test Results.

Method	CS↑	CV1↑	CV2↑
HAM-HGNet [24]	68.32	72.15	70.88
KDS-GCN [26]	75.28	78.44	76.92
CBAM-STGCN [25]	73.89	76.12	74.55
Wu et al. [27]	76.55	79.01	77.33
SkateFormer [29]	77.12	79.85	78.20
ASTM-Net-ALLs	**79.34**	**82.17**	**80.45**

As shown, ASTM-Net achieves 79.34% on CS and 82.17% on CV1, surpassing Wu et al. [27] by 2.79% and 3.16%, respectively. This validates its ability to handle occlusions (e.g., hands behind objects) and viewpoint shifts through dynamic 3A-Graphs. In CV2, ASTM-Net attains 80.45%, outperforming SkateFormer [29] by 2.25%, demonstrating robustness to mixed-view training and front-view testing. However, we found some failure cases, e.g., activities like “handing objects” achieve only 71.3% accuracy due to limited inter-person relationship modeling. sequential occlusion of critical joints (e.g., hip nodes) in long-duration activities (“clean dishes”) leads to ~8% accuracy drop. This is also our future direction of improvement.

### H. Visualizations

**Fig 8:**
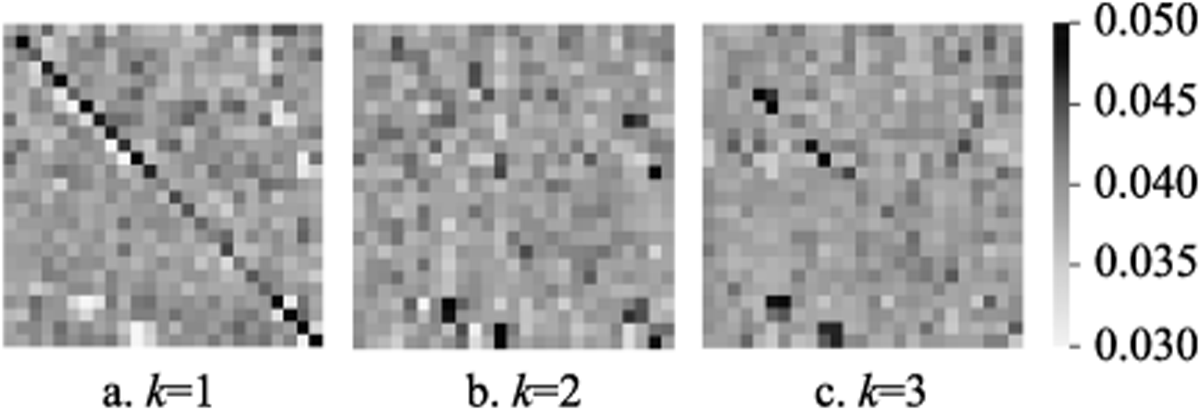
The visualizations of skeletal adjacency graph matrices without the activity-related graph.

Taking the activity “jumping” as an example, Fig 8 illustrates the skeletal adjacency graph matrices without the optimization of the activity-related graph *C*_*k*_, represented by the sum of the initialized matrix *A*_*k*_ and the trainable matrix *B*_*k*_ for the three spatial subsets (*k* = 1,2,3). Figs 8a-8c visualize *A*_1_ + *B*_1_, *A*_2_ + *B*_2_, and *A*_3_ + *B*_3_, respectively. In the visualization, darker color blocks indicate stronger node affinities, while lighter colors represent weaker relationships.

The results show that:

1) In the first spatial subset, the learned adjacency graph focuses more on self-connections (indicated by the concentration of dark blocks along the diagonal). In the second and third spatial subsets, the topology focuses on the affinities between different nodes.
2) The learned adjacency graph can capture non-physical node affinities.
3) The adjacency graphs differ across the three spatial subsets, providing rich features for spatial graph convolution.

**Fig 9:**
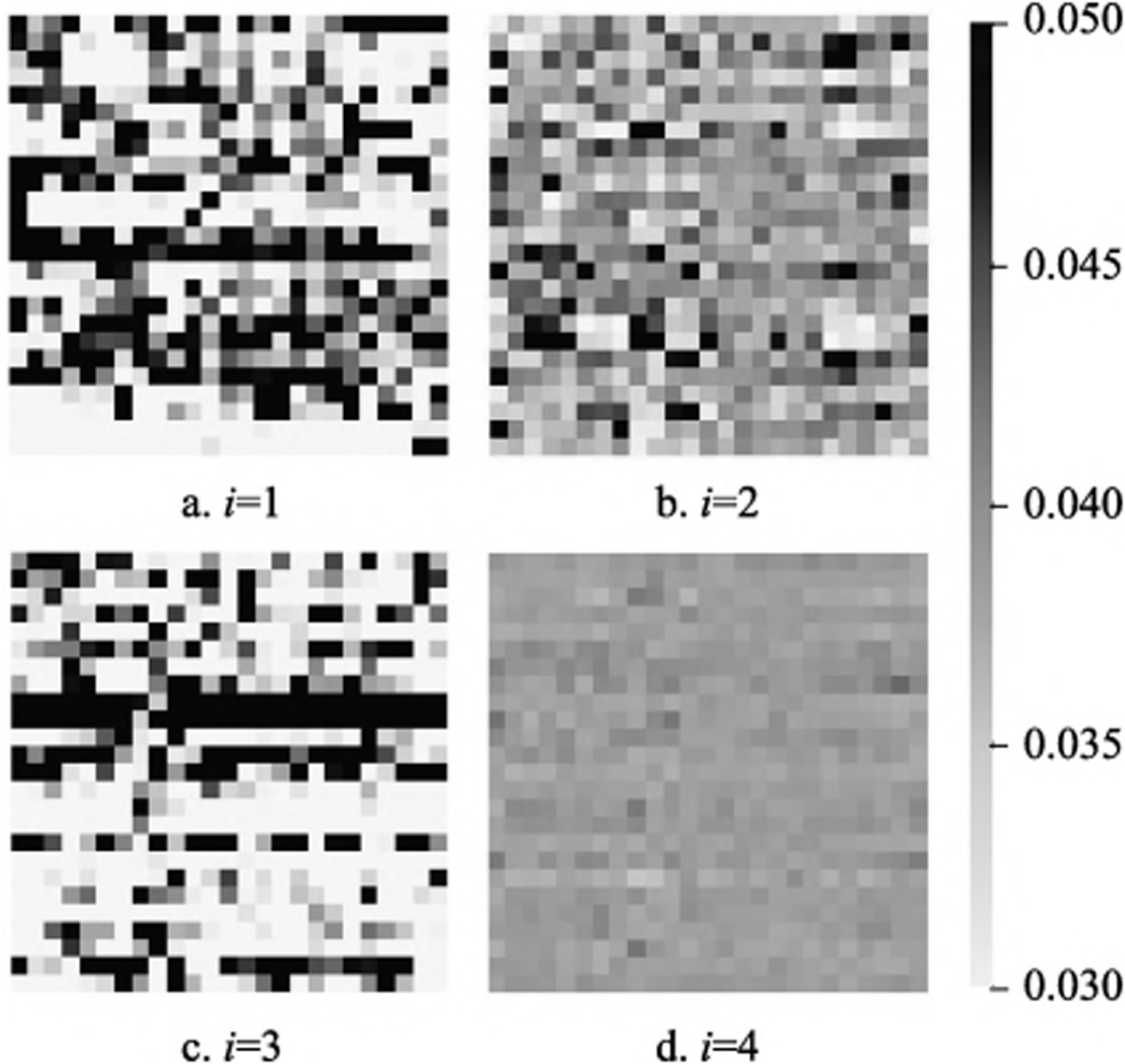
The visualizations of the activity-related graphs.

Fig 9 further visualizes the activity-related graphs *C*_*ki*_ at different time periods within the same spatial subset, still using the “jumping” activity as an example. As described in the methodology section, *C*_*k*_ is obtained by concatenating *s* activity-related graphs, with *s* = 4 as determined in last section. In Fig 9, $C_{ki}\in R^{N*N},i=1,2,3,4$ represents the activity-related graph at different time periods. Figs 9a-9d show the visualization results for *C*_*k*1_, *C*_*k*2_, *C*_*k*3_, and *C*_*k*4_, respectively.

The results demonstrate:

1) The activity-related graphs differ from the unoptimized graph, enriching the spatiotemporal features of the nodes.
2) Activity-related graphs vary across different time periods, verifying the necessity of optimizing node affinities dynamically over time.

**Fig 10:**
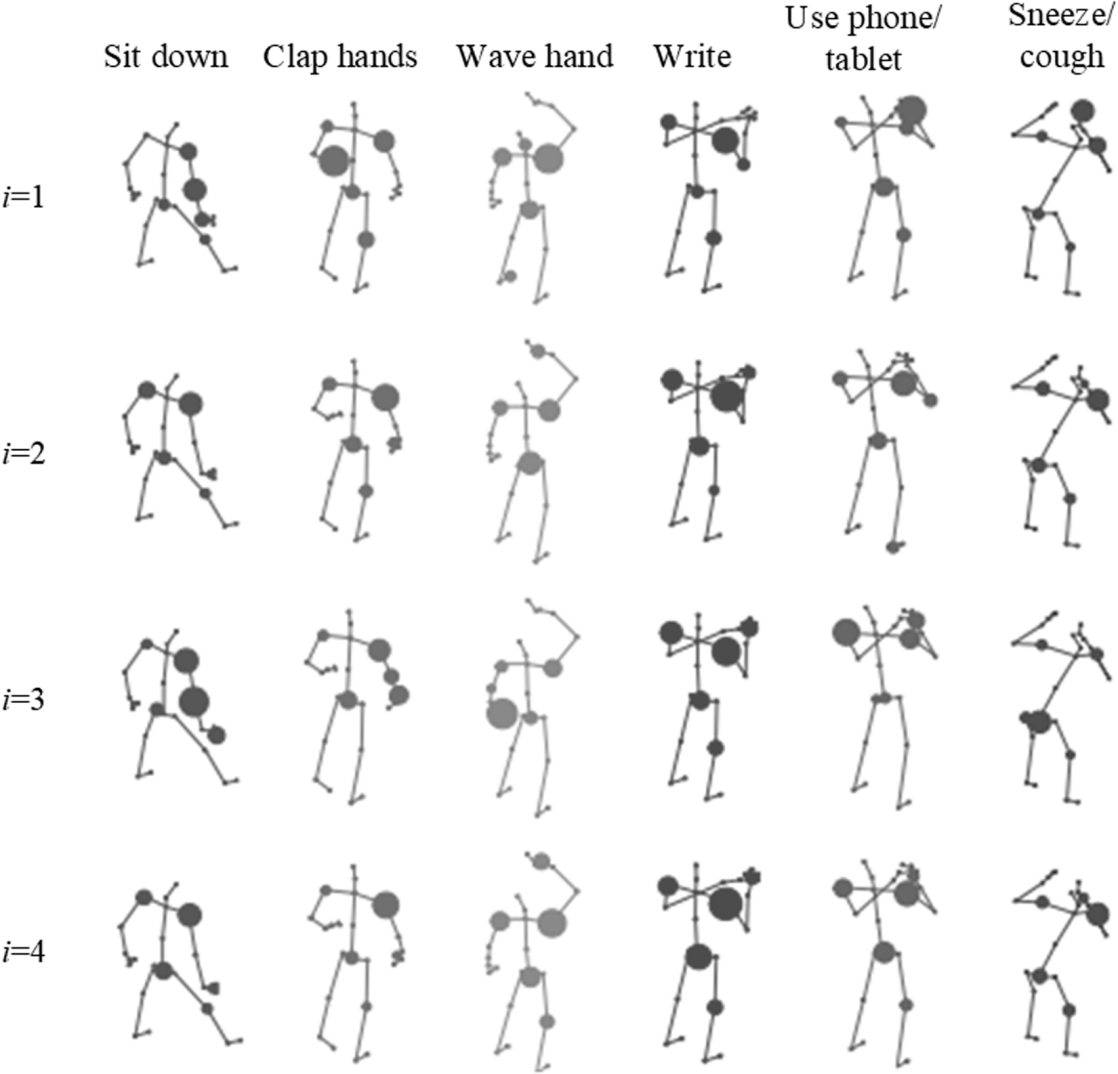
The 3A-Graph3A-graph visualizations with different settings.

Fig 10 visualizes the final learned skeletal adjacency graphs $G_{ki},i=1,2,3,4$ for different activities at different time periods. All graphs are derived from the same convolutional layer and spatial subset of ASTM-Net. Specifically, each circle represents a node, with its size indicating the connection strength between the current node and the 25th node (the hand node). Only the top 5 strongest connections are visualized.

The results show:

1) For both coarse activities (e.g., “sitting,” “clapping,” “waving”) and fine-grained activities (e.g., “writing,” “using a phone/tablet,” “sneezing/coughing”), the learned skeletal adjacency graphs differ across time periods.
2) For different activities with highly similar activity trajectories (e.g., “writing” and “using a phone/tablet”), the learned adjacency graphs are still distinguishable.

These findings validate our hypothesis: different samples with distinct activity patterns require different adjacency graphs to capture node affinities, and optimizing skeletal graphs with activity-related graphs over time is necessary.

## V. Discussion

### a. Novel added value

Beyond advancing state-of-the-art accuracy, ASTM-Net introduces two paradigm-shifting values that redefine efficiency and adaptability in skeleton-based HAR:

#### 1 ) Dynamic 3A-Graphs with Activity-Conditioned Optimization.

Prior works either use fixed physical graphs [19] or learn global attention weights [25], neglecting the temporal dependency of node relationships. Our 3A-Graph framework innovates by:

a) Decomposing graph learning into stable (physical/trainable graphs) and dynamic (activity-related graph) components;
b) Conditioning spatial connections on localized temporal features through segment-wise similarity learning (i.e., (8));
c) Optimizing graphs via parameter-shared inference across time segments (*s* = 4), reducing parameters versus layer-specific alternatives [23].

#### 2) Parameter-Efficient Multi-Branch Temporal Modeling.

TMGM redefines temporal convolution through heterogeneous branches that collaboratively capture:

a) Short-range dynamics (*d*=1 branch: 5-frame micro-motions);
b) Long-range dependencies (*d*=3 branch: 13-frame macro-trends);
c) Noise resilience (pooling branch: suppressing transient outliers);
d) Feature preservation (1*1 branch: maintaining raw signal fidelity);

Unlike transformer-based methods [29] that compute O(*T*²) attention weights, TMGM’s branched convolutions operate at *O*(*T*) complexity, achieving lower FLOPs than MST-GCN [54] while improving accuracy (as shown in Table 3).

### b. Future work

This section evaluates the performance of ASTM-Net under different hyper-parameter settings, including the reduction ratio *r* and the number of feature segments *s* (i.e., the number of time periods). The results are presented in Table 6.

While ASTM-Net achieves state-of-the-art performance in skeleton-based activity recognition, several promising directions remain to furtherenhance its applicability and scalability in real-world scenarios. Below, we discuss potential extensions and improvements:

#### 1) Integration with Transformer Architectures.

The success of Transformers in modeling long-range dependencies suggests opportunities to enhance ASTM-Net’s temporal modeling capabilities. Replacing TMGM’s dilated convolutions with lightweight self-attention mechanisms could better capture global temporal patterns (e.g., periodic motions in “jumping rope”). Combining ASGM’s dynamic graphs with Transformer encoders may further exploit local-global spatiotemporal dependencies. To validate this, future work could compare the original ASTM-Net with transformer-enhanced variants such as ASTM-Transformer (replacing TMGM with vanilla multi-head self-attention) and ASTM-EfficientFormer (hybrid sparse attention + dilated convolution) on NTU-RGB + D and Toyota Smarthome datasets. Key metrics would include accuracy, FLOPs, and inference speed on edge devices like NVIDIA Jetson. Implementation steps could integrate shifted window attention to reduce quadratic complexity and apply dynamic token sparsification to prune non-salient frames. Quantizing attention weights to INT8 would further optimize edge deployment. Evaluation would focus on accuracy-FLOPs trade-offs and interpreting attention maps for critical motion phases. Challenges such as computational overhead could be mitigated via low-rank approximations or hardware-aware optimizations.

#### 2) Self-Supervised Learning for Label Efficiency.

Current methods rely on fully annotated datasets, limiting scalability. Self-supervised pretraining via masked joint reconstruction or contrastive learning could improve label efficiency and robustness. For instance, pretraining on PKU-MMD V2 (5.4M unlabeled frames) with strategies like masking 40% of joints/frames (L1 reconstruction loss) or generating positive pairs via temporal cropping/jittering could enable robust feature learning. Downstream fine-tuning on NTU-RGB + D (10%/30%/100% labeled data) and Toyota Smarthome would validate label efficiency gains. Success metrics would include achieving >85% accuracy with 10% labels (vs. 70% supervised baseline) and improved robustness to occlusions (RND/CFO metrics). Visualization of pretrained embeddings via t-SNE could verify activity clustering. Cross-modal alignment with CLIP embeddings, if RGB data is available, might further enhance generalization to unseen domains like medical rehabilitation.

#### 3) Privacy-preserving HAR via federated learning.

For sensitive applications like healthcare or smart homes, federated learning (FL) can enable collaborative model training without sharing raw skeleton data. One promising direction is extending ASGM to learn personalized 3A-Graphs on distributed devices while aggregating only graph parameters, avoiding the need to transmit raw data. Another avenue is injecting noise into graph updates during FL to prevent inference attacks on user-specific motion patterns, thus ensuring privacy. Furthermore, optimizing ASTM-Net’s architecture for varying computational capacities (e.g., smartphones vs. IoT sensors) in FL frameworks could further enhance its adaptability.

#### 4) Multi-modal fusion for complex scenarios.

While ASTM-Net focuses on skeleton data, integrating complementary modalities could address its limitations in occlusion-heavy environments. RGB-skeleton fusion using attention-based modules could combine skeletal features with RGB appearance cues to improve performance in scenarios like object interactions (e.g., “open fridge”). Depth maps can refine node distances in the 3A-Graph, improving robustness to viewpoint changes. Additionally, for activities with sound cues, such as “clapping” or “typing,” synchronizing audio features with skeletal motions via cross-modal transformers could enhance recognition.

#### 5) Edge deployment and hardware optimization.

To realize real-time HAR on resource-constrained devices, future work should explore quantization and pruning techniques. Post-training quantization could reduce ASTM-Net’s precision (FP32 → INT8), while pruning redundant graph connections would help to decrease model size and improve inference speed. Hardware-aware neural architecture search (NAS) could be employed to automatically design ASTM-Net variants optimized for specific hardware chips like NVIDIA Jetson or ARM CPUs. Furthermore, enabling incremental learning on edge devices could allow for adaptation to user-specific motion patterns without requiring cloud dependency.

#### 6 ) Real-world deployment and industrial validation.

Although ASTM-Net achieves promising performance on benchmark datasets such as Toyota Smarthome, its practical deployment in real-world scenarios—especially in embedded systems like wearable devices and smart surveillance systems—remains a critical next step. For wearable device integration, hardware-specific optimization is necessary to adapt ASTM-Net to ultra-low-power ARM processors (e.g., Cortex-M7), involving pruning redundant graph connections and employing INT8 quantization for reduced power consumption. Comprehensive energy efficiency evaluations using commercial wearables such as Fitbit Sense and Apple Watch under continuous activity monitoring conditions would further ensure practical viability. Additionally, enabling on-device fine-tuning could facilitate personalized models capable of adapting to individual-specific motion patterns, particularly beneficial in medical scenarios like detecting gait abnormalities in Parkinson’s patients. For smart surveillance applications, ASTM-Net can be enhanced by incorporating multi-camera fusion strategies to robustly handle occlusions in crowded environments, leveraging synchronized skeleton streams from distributed camera systems. An edge-cloud hybrid deployment—deploying lightweight ASGM modules at edge devices for instantaneous alerts such as fall detection, while conducting deeper multi-scale TMGM analyses on cloud platforms—could balance responsiveness with computational efficiency. Moreover, adopting federated learning methodologies would enable privacy-preserving inference, anonymizing sensitive skeletal data across distributed surveillance nodes to ensure compliance with GDPR and CCPA regulations. Finally, establishing industrial partnerships, such as collaborations with healthcare providers like Philips Lifeline to validate the system’s reliability in elderly monitoring or integration with smart home manufacturers such as Bosch or Nest for real-time IoT activity recognition, would significantly facilitate the practical adoption and validation of ASTM-Net in real-world applications.

## VI. Conclusion

This paper introduced ASTM-Net, a novel framework for skelecton-based activity recognition that integrates spatial and temporal information through the proposed ASGM and TMGM modules. By dynamically modeling 3A-Graphs, ASGM effectively captures the temporal variations in node affinities and enhances the robustness of spatial graph convolutions. The incorporation of TMGM further strengthens the temporal modeling capacity by extracting multi-scale features. Experimental results on different datasets highlight the superior performance and efficiency of ASTM-Net, achieving state-of-the-art accuracy with significantly reduced parameters and computational costs compared to existing methods. Ablation studies validate the contributions of ASGM and 3A-Graphs to the overall performance. In conclusion, ASTM-Net provides a lightweight yet powerful solution for skeleton-based activity recognition, paving the way for future research into dynamic graph modeling for HAR. In addition, while evaluated on general daily activities, the proposed ASTM-Net can be potentially applied to sports scenarios (e.g., analysing athlete movements) due to its capability in modelling dynamic joint correlations. Future work includes validating ASTM-Net on sports-specific datasets (e.g., basketball tactical analysis) to further explore its robustness in high-dynamic scenarios.
